# A Novel Approach of Promoting Adhesion of Reinforcing Cord to Elastomers by Plasma Polymerization

**DOI:** 10.3390/polym11040577

**Published:** 2019-03-29

**Authors:** Wilma Dierkes, André Louis, Jacques Noordermeer, Anke Blume

**Affiliations:** 1 University of Twente, Dept. of Mechanics of Solids, Surfaces and Systems, Chair of Elastomer Technology and Engineering, P.O. Box 217, NL-7500 AE Enschede, The Netherlands; Andre.Louis@apollotyres.com (A.L.); j.w.m.noordermeer@utwente.nl (J.N.); a.blume@utwente.nl (A.B.); 2 Apollo Tyres Global R&D B.V., Ingenieur Schiffstraat 370, NL-7547 RD Enschede, The Netherlands

**Keywords:** adhesion, elastomer, rayon, polyethylene terephthalate, PET, tire, RFL, plasma, silane

## Abstract

Adhesion of cords to elastomers is crucial for many elastomeric products, such as tires and V-belts. The best adhesion system so far is based on a combination of resorcinol, formaldehyde, and a latex (RFL). However, this cord treatment has serious disadvantages in terms of processing and toxicity. A promising alternative is a plasma treatment of the cords prior to be embedded in the elastomer. For rayon cords, a plasma polymerization of sulfur-containing precursors results in adhesion levels close to RFL treatment. However, for polyethylene terephthalate (PET) cords, this treatment is not satisfactory. For this type of cords, a water-plasma activation followed by a silane dip is more promising, as 72% of the adhesion level of RFL treatment could be achieved. For rayon, an even higher adhesion level was realized.

## 1. Introduction

Polymeric fibers are widely used in the rubber industry, e.g., in tires, conveyor belts and V-belts, as they add strength and form stability to the elastomer matrix. However, in order to achieve the necessary reinforcement, good adhesion between the polymeric fiber and the matrix has to be guaranteed. 

The most commonly used technology to achieve adhesion between the rather apolar elastomers such as natural rubber (NR), styrene-butadiene rubber (SBR), and butadiene rubber (BR) is the resorcinol-formaldehyde-latex (RFL) dip, applied to the fiber before being built into the elastomer product. This adhesion system is the industrial standard for fiber-reinforced elastomers since 1938 [1]. The RF resin provides bonding to the cord, and the elastomer-like latex component bonds to the rubber by co-vulcanization. A thorough investigation of the mechanisms of this adhesion system was done by Wennekes et al. [2,3,4]. The term RFL-treatment covers a large variety of treatments; they are based on the same technique but differ in recipe and application. This implies that the system is adjustable for various combinations of materials used for specific composites, as each cord and elastomer may require optimized treatment conditions.

**Scheme 1:**
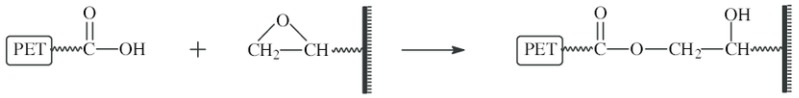
Schematic reaction pattern of an epoxy coating with a polyethylene terephthalate (PET) end group [9].

Tires are the largest market for long fiber reinforcement, with polyamide and polyester representing the highest quantities, followed by rayon [5]. The adhesion is in general based on chemical bonding at the interface between dip and cord, but mechanical and physical interactions contribute as well to the overall adhesion effect [6]. Polyethylene terephthalate (PET) cords, which belong to the polyesters, are much less reactive and an additional treatment is mandatory to obtain sufficient adhesion. The reason for the low reactivity is the chemical inertness of polyester, which results in a poor compatibility with and wettability of the RFL adhesive coating [7]. To achieve adhesion between both components, an extra treatment of the cord is necessary in order to provide compatibility. Most common is an epoxy pre-treatment of the polyester cord: a sub-coat between the cord and actual RFL-coating is applied [8], which reacts with the polyester cord as shown in Scheme 1, and activates it for further interaction with the RFL-dip. 

In general, RFL-dip systems deliver very good results for adhesion promotion between elastomers und reinforcing cords. That is the major advantage of this technique, but it also has some drawbacks, and these are the driving forces to substitute the RFL-dip treatment by alternative treatments. The main drawbacks are the multistep processing, which is costly and requires time and effort, as well as the environmental burden and health aspects [10]. Even though nowadays industrial setups provide high protection standards for workers and the environment, the residues from the RFL-dip processing often must be handled as special waste. The health concerns are mainly regarding evaporation of resorcinol during production and application of the dip. Resorcinol has a hazardous nature and is considered dangerous to the environment. In addition to resorcinol, formaldehyde is also problematic, as it is highly toxic to all living beings, regardless of the method of intake, and is confirmed to be possibly carcinogenic (class 1B) [11]. Alternatives to RFL treatment are resorcinol- and formaldehyde-free dips [12,13], as well as cord materials with moieties which can directly react with the elastomer [14], making dipping or other treatments unnecessary.

Plasma technology to replace the RFL treatment is another alternative. Initially, this method was used to enhance the adhesion of the standard RFL treatment. One of the first patents in this field was filed by Lawton et al. [15] and dates back to 1974. The topic is “Bonding of Poly(Ethylene Terephthalate) Induced by Low-Temperature Plasmas” and claimed is a low-pressure plasma reactor which batchwise treats polyester in a non-oxidizing plasma. Sharma et al. claimed a similar patent for the adhesion of aramid cords to rubber in 1984 [16]: An aramid cord is treated with plasma of air, N_2_, He, Ne, or Ar or a mixture thereof, followed by a RFL dipping. In this case, the adhesion was increased by up to 50%; for un-dipped cords the adhesion was improved by up to 30%. Several patents are filed by Goodyear: In 1991, a gas plasma process using oxygen and tetrafluoromethane was claimed for activating the fiber surface prior to the dipping process [17]. Other patents describe the use of a polymerizable precursor such as carbondisulfide [18,19]. In another approach filed in 1996, reactive groups containing active hydrogen were bonded to a polymeric cord surface. As processing gas NH_3_ and CF_4_/O_2_ mixtures were used. The reactive hydrogen groups can later react with blocked isocyanates at elevated temperatures to form a chemical bond to the elastomeric rubber network [20]. Another alternative is the introduction of vinyl groups on the cord surface followed by a RFL treatment. A special plasma polymerization nozzle developed by Goodyear protects the plasma activated cord by the plasma gas until it gets into contact with the precursor material. This eliminates potential disturbances from the environment and thus prevents unwanted reactions [21].

Other patents [22,23] describe a three-step process: 

(a) Atomizing a mixture of at least one polymerizable monomer, a halogenated saturated hydrocarbon, an optional curative and a carrier gas to form an atomized mixture; 

(b) generating an atmospheric pressure plasma from the atomized mixture; and

(c) exposing the reinforcement cord to the atmospheric pressure plasma under conditions suitable to form a polymer strongly bonded to the tire cord and capable of bonding to rubber.

The application claims a variety of chemicals that can be used to create an atomized mixture. As possible carrier gases are named argon, helium, neon, xenon, oxygen, nitrogen, and carbon dioxide. Other gas mixtures mentioned for adhesion enhancement contain a sulfur compound and an alkyne [24].

A method for treating and modifying the surface of a polyester fiber was disclosed by Ueno [25]. The plasma treatment using helium resulted in fibers with highly oriented molecules and high crystallinity, tenacity, and modulus of elasticity. Additionally, they exhibit an improved adhesion to an epoxy matrix. Hudec [26] also used plasma treatment to enhance the adhesion of polyester to rubber. In this plasma setup, the cord runs through the center of the electrodes while being protected by a nitrogen atmosphere that is present in the glass chamber. Adhesion values close to the ones of RFL-coated polyester fibers were reported.

Patent WO 2006/135347 [27] by Janypka et al. claims a method of treating a textile material by low temperature plasma at atmospheric pressure. Nitrogen is used as carrier gas and additionally a mixture of propane and butane is fed into a plasma chamber, leading to the deposition of a polymer layer on the surface of the treated textile. The method is claimed for (pulsed) corona and coplanar dielectric barrier discharge. The adhesion strength of the treated PET cords compared to standard RFL treatment shows good adhesion values and proves that the method is working. The patent from Mihalik [28] covers a device for manufacturing rubberized fabric cord including a plasma treatment, that is suitable to be used as wound overlapping breaker in tire construction and has a positive effect on the tire properties. 

Silanes are commonly used for steel/rubber adhesion, and are also reported in literature as precursors in a plasma treatment process [29]. They are also used for better adhesion between silica as a reinforcing filler and rubber since Michelin introduced the green tire concept in 1992 [30]. Silica and elastomers lack compatibility and, thus, appropriate adhesion, as they differ considerably in polarity and reactivity. What brings these two components together is the use of a coupling agent: a bifunctional molecule with a moiety to react with the polymer and another reactive group which forms a bond to the silica surface. 

This approach can also be applied to a reinforcing cord. However, the low reactivity of fibers like polyester requires a highly reactive treatment system. Using plasma treatment, the deposition of a silica coating onto the cord surface is possible. As precursor, tetraethyl orthosilicate, also known as tetraethoxysilane (TEOS), is used. The latter is commonly used in industry as a silica precursor for coatings and in aerogels.

**Scheme 2:**
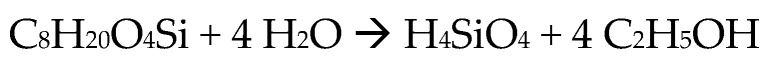
Hydrolysis reaction of TEOS.

TEOS is the reaction product of silicon tetrachloride and ethanol and has a tetrahedral molecular structure due to the centered silicon atom. It is insoluble in water, but very well soluble in ethanol. Adding water to TEOS, it undergoes a hydrolysis reaction as shown in Scheme 2. Under neutral conditions, this is a very slow reaction, which can be accelerated by either acidic or alkaline conditions:

The reaction shown in Scheme 2 is a hydration process, which liberates ethyl alcohol. The intermediate product can be further condensed to form an oligomeric pre-polymer, which is often used in coatings, or converted further to silicon oxide, SiO_2_. The creation of monodisperse SiO_2_ nanoparticles is possible with a good control over the particle size. The control parameters are concentration, temperature and ammonia content, and the particle size is in a range of 20–500 nm.

TEOS can be applied in a plasma process in two ways: It is either used in its pure form, or, as described above, in a mixture with ethanol and a certain amount of distilled water. The first option takes the scission of the molecule in the plasma into account. A recombination of the TEOS fragments on the cord surface can create silica-like structures while still featuring ethoxysilanes. In the latter option, next to interaction with the plasma, a hydrolysis reaction can also be expected when exposing the mixture to plasma. Both, the creation of an oligomeric pre-polymer as well as of monodisperse silicon oxide particles can potentially occur.

In the next step, the challenge is to find a proper procedure to introduce the coupling agent and to let it react with the silica deposit on the cord surface. It requires a temperature of about 140 °C: high enough to let the silanization reaction take place at a reasonable rate, but still low enough to avoid degradation of the elastomer. This reaction has to happen either before the cord is embedded in the elastomer matrix, or during the curing step of the final product.

In this study, a plasma treatment for the deposition of a silica layer onto the cord surface for both, rayon and PET cords, is developed, and different solutions for the incorporation of the coupling agent are explored. Additionally, another alternative plasma treatment to the silica coating is investigated.

## 2. Materials and Methods 

From an experimental point of view, the plasma activation of the cords involve several steps. These steps are as follows:

- Plasma treatment (with precursor)
- Dipping of the activated cord with the coupling agent
- Controlled drying of the dipped cord
- Co-vulcanization of dipped cord and rubber

**Figure 1:**
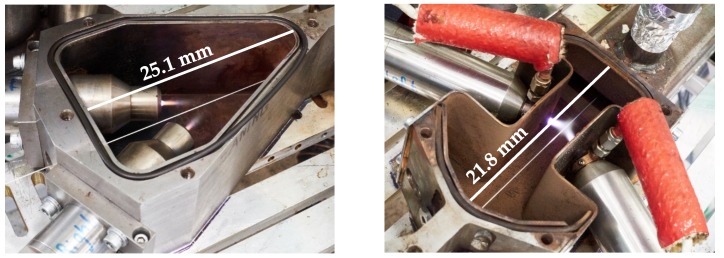
**Left**: decontamination chamber; **right:** plasma coating chamber.

*Plasma reactor:*Figure 1 shows the core part of the plasma modification reactor, consisting of a cleaning and a polymerization unit. These two separate chambers are each equipped with two atmospheric pressure plasma jets (APPJ). The two plasma jets within one chamber are positioned horizontally in order to have a homogenous modification around the cord. The chambers are continuously flushed with nitrogen and kept under slight overpressure to initially remove oxygen and keep the cord under a protective gas atmosphere. A single cord is guided horizontally through the chambers and an extension, prolonging the residence time of the cord in the plasma gas zone.

The plasma treatment of the cord was performed with a decontamination step using nitrogen, directly followed by the plasma coating step. This was done in an air plasma using filtered compressor air as the ionization gas. The precursor carrier gas was also nitrogen. A voltage of 278 V and a current of 5.8 A were applied for the initial cleaning step.

A general problem of plasma treatment is the thermal burden for the cord, which can change the cord properties. Therefore, it is crucial to thoroughly control the cord temperature during plasma treatment. Another issue is the short lifespan of the plasma-activation. This makes it necessary to initiate the reaction with the polymer as fast as possible after the plasma treatment. In the setup used within this study, a homogenous coating of the cords is another challenge. To avoid a shadow side, two APPJs were used as shown in Figure 1. However, even this arrangement of the APPJ’s very probably does not result in a homogenous coating over the whole outer surface of the cord. The penetration of the cord by the activated precursor is yet another issue. Another aspect is the ageing resistance of the final composite material. Non-published tests have shown, that the effect of thermal aging is independent from the treatment: plasma- and RFL-treated cords show similar ageing behavior. 

*TEOS treatment:* The plasma coating settings for the TEOS treatment were as follows: the precursor flow rate was set to 150 g/h and the evaporation temperature to 200 °C for all experiments. For the plasma generator itself, the settings given in Table 1 were used.

These plasma settings were determined in prior experiments and represent the upper limit values, thus the most powerful plasma to be run. The cord speed, thus the treatment time was also determined in prior experiments. The limiting factor for the low speed side, that is long treatment times, is the thermal burden on the cords, which would change its properties. Therefore, the cord speed was limited to 10 meters per minute (m/min). 

*Water vapor treatment:* The activation of rayon as well as PET cords by water vapor was done using the following parameters:

- Evaporation temperature: 110 °C
- Pre-drying of cord: no
- Flow rate (FR): 300 g/h, 400 g/h
- Ionization gas (ION): air, nitrogen

The evaporation temperature of 110 °C was chosen, as it is above the boiling temperature of water and thus generates a sufficient high water vapor pressure. Pre-drying of the cord did not have any influence on the properties of the treated cord and was therefore not necessary. The flow-rates of the precursors were chosen to be as high as possible (400 g/h), and significantly lower but still high enough to expect a considerable degree of coating (300 g/h).

**Figure 2:**
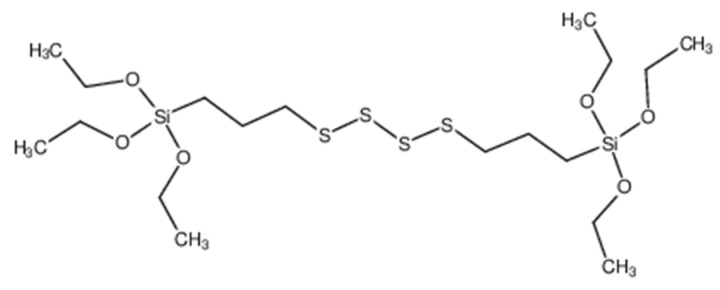
Silane-coupling agent TESPT (bis-[3-(triethoxysilyl)propyl]tetrasulfide).

*Reaction of the cord with a silane:* As coupling agent, a commonly used silane, bis-[3-(triethoxysilyl)propyl] tetrasulfide (TESPT), was used (see Figure 2). The triethoxysilyl groups, which are located on both ends of the molecule, will react with the silanol groups of the silica on the modified cord surface. In this reaction, siloxane bonds are formed in a primary reaction step under generation of ethanol as side product [31,32]. The polysulfane chain segment in the middle of the molecule with an average sulfur chain length of 3.8 (actually a mixture of sulfanes with different sulfur chain length) is not temperature stable. It breaks apart at elevated temperatures during vulcanization. This liberates free sulfur and creates two highly active bonding sites for the cross-linking sulfur complexes formed during the vulcanization reaction. These react with the TESPT fragments and with the rubber chains. This reaction mechanism creates a bonding of the SiO_2_ plasma coating on the cord via TESPT to the rubber matrix. This way, the system provides a chemical bond between cord and rubber.

**Figure 3:**
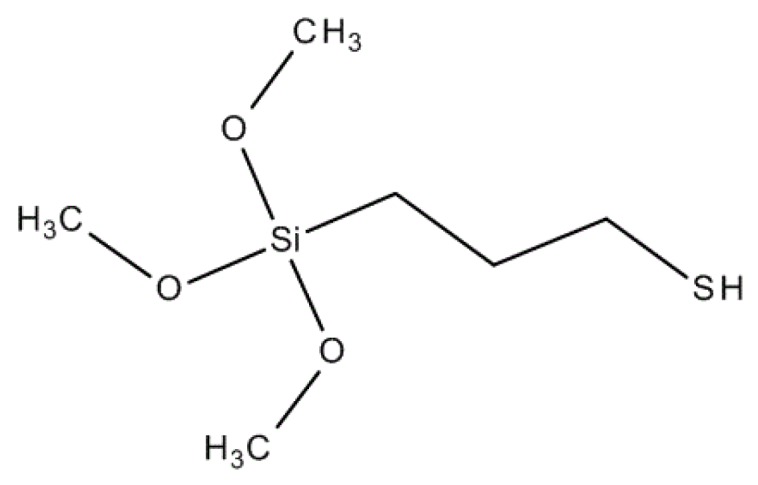
Silane-coupling agent MPTES (mercapto-propyl-triethoxysilane).

As an alternative to TESPT, a second coupling agent, mercapto-propyl-triethoxysilane (MPTES), was used for comparison. It features only one triethoxysilyl group and instead of the sulfur chain segment, it has a highly reactive mercapto group (S-H), that terminates the molecule on the other side (see Figure 3).

Two different methods were studied to incorporate the coupling agents:

- Incorporation of the coupling agent directly into the rubber as an ingredient of the compound. This was done on a two-roll-mill using the reference compound given in Table 2. As the required amount of coupling agent for a successful cord-to-rubber bonding was unknown, two concentration levels were used: 3.7 and 9.2 parts per hundred rubber (phr). A disadvantage of this method is that the silanization process has to occur in the rubber phase during vulcanization: ethanol as side product is generated and cannot evaporate under these circumstances. This may cause porosity later on and may even affect the adhesion values.
- A dipping bath containing the coupling agent was used to deposit the latter onto the TEOS coated cord. Subsequently, after the dipping step a drying step in an oven was required. While drying the cord, the silanization reaction can take place and ethanol as reaction byproduct can easily evaporate. The previously mentioned disadvantage of having ethanol residues trapped in the rubber is, therefore, avoided. Afterwards, the so-prepared cord was vulcanized together with the unmodified reference rubber compound given in Table 2. The initial dipping was done with the Bath I composition, which was prepared according to the recipe shown in Table 3. As buffer, either acetic acid or triethylamine were used, as the entire process would run very slow under pH-neutral conditions. The process is sensitive to the concentration of the coupling agent, as high concentrations can cause TESPT oligomerization instead of a reaction with the silanol groups on the cord surface. The dipping time was 60 minutes at room temperature in a closed embodiment.

**Figure 4:**
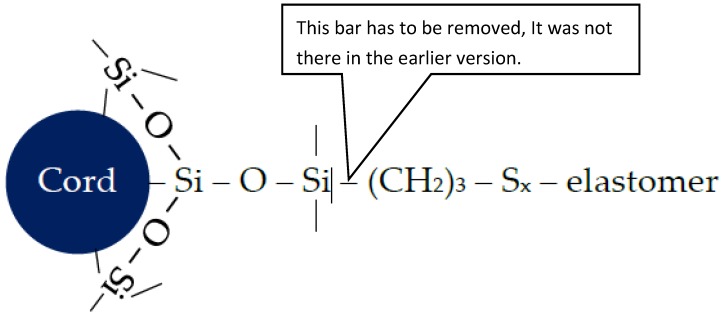
Bond between the cord and elastomer.

*Drying process*: To finalize the TEOS coating and coupling agent dipping step, it is necessary to activate their chemical reaction via heat. The cords were taken from the dip and allowed to dry at room temperature for 10 minutes under tension. This is beneficial, as the cords get stiffer. All cords were dried under tension as well in an oven at 100 °C for 15 min. The cords were then embedded between two rubber layers and cured in the press at 160 °C for 90% of the optimum curing time, t_90_. All these subsequent steps result in a strong covalent bond between the cord surface and the polymer via the bifunctional silane molecule as illustrated in Figure 4. 

The dipping bath composition and treatment conditions for the water vapor activated cords were further optimized in a design of experiments (DoE) approach. The following parameters were chosen: A-water content; B-TESPT/acid content; C-temperature/time. The settings of the parameters were:

- A+ = 9 mL            A− = 1.8 mL
- B+ = 4.35 mL TESPT, 4 mL acid   B− = 0.87 mL TESPT, 1 mL acid
- C+ = 120 °C, 2 min        C− = 60 °C, 120 min

The values of these parameters were higher and lower than the initial parameters elaborated in earlier experiments. The plus and minus sign signify the lower and higher settings of these parameters relative to the experimental settings of these initial experiments. 

*Adhesion measurements:* The adhesion between cord and elastomer matrix was tested in an H-pullout test. This test method is applicable to all textile cords, based on natural, as well as synthetic fibers. The test sample is H-shaped, and the connection between the two parallel bars made from the elastomer material is the cord itself. The force to pull the cord out of the bars is measured. It is documented in ASTM standard D4776M-10. 

## 3. Results

### 3.1. Rayon Cord

**Figure 5:**
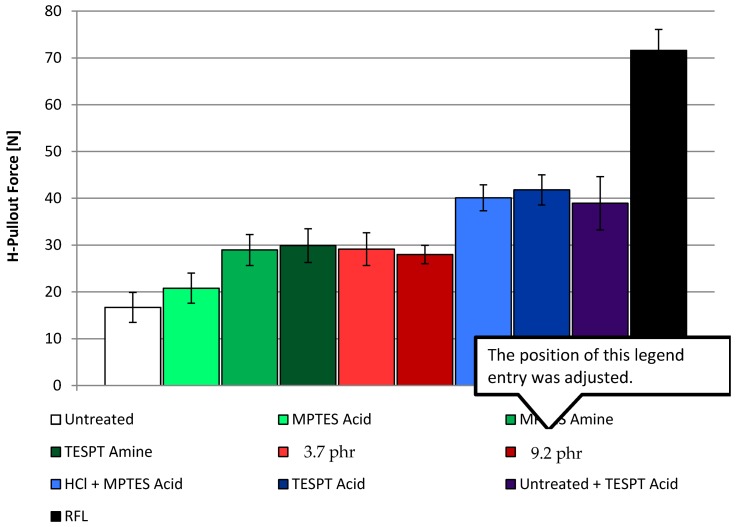
H-Pullout results for TEOS-plasma-coated rayon cords.

The experiments illustrated in Figure 5 were done under the following plasma treatment settings, which were found to give a stable plasma and efficient coating:

- Treatment speed: 10 m/min
- TEOS flow rate: 150 g/h
- Evaporation temperature: 200 °C
- Pre-drying of cord: no

The untreated rayon cords did show the lowest adhesion (16.7 N). Dipping the cords in a MPTES/acid mixture (type Bath I) had nearly no effect as the adhesion reached 20.8 N, while using the MPTES/amine mixture resulted in an improvement of the adhesion to 29 N. Almost the same result with 29.9 N was achieved with a TESPT/amine dip (type Bath I). 

When TESPT was incorporated into the matrix compound, the adhesion levels were similar to the one measured on the Bath I type test specimens: With 3.7 phr TESPT incorporated into the compound, the H-pullout force was 29 N, and with 9.2 phr it was 28 N. That indicates that 3.7 phr of TESPT in the rubber compound were sufficient to make full use of the hydroxyl groups present on the cord surface after the plasma treatment. 

To see if the plasma deposition itself was inadequate, or if interaction with the coating is missing due to a lack of hydroxyl groups, the following measures were taken:

- Repetition of the Bath I type dipping with a small amount of hydrochloric acid (HCl) added. HCl donates H+ ions, which can react with oxygen to increase the number of available OH groups.
- Dipping the cord in Bath II with an increased amount of demineralized water and acetic acid, which is expected to increase the number of available OH groups.

Compared to the previous results, the adhesion increased roughly by 10 N with the adjusted methods (blue bars). This supports the assumption that more OH groups are necessary to increase the number of siloxane bonds and, thus, the adhesion strength. However, using an untreated cord in the as-delivered state and just dipped in a type II bath, see Figure 5: Untreated + TESPT/acid, nearly gave the same results as a plasma coating, see the same Figure: TESPT/acid. However, the standard deviation was higher for the untreated cord. This indicates, that the plasma harmonizes the cord surface to a certain degree allowing more steady results in mechanical tests. However, it also shows that the plasma deposition of TEOS under these processing conditions was not successful for rayon cords. 

### 3.2. Polyester Cord 

**Figure 6:**
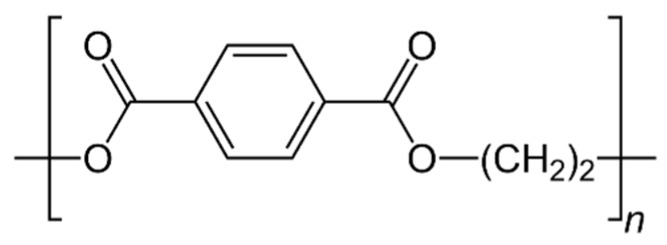
Structure of the polyester backbone.

The backbone of polyester does not contain any hydroxide groups as shown in Figure 6, and the cord itself does not take up moisture from the environment. These properties differ from the ones of rayon cord and decrease the possibility of forming OH groups on the cord surface when applying a TEOS plasma coating. 

**Figure 7:**
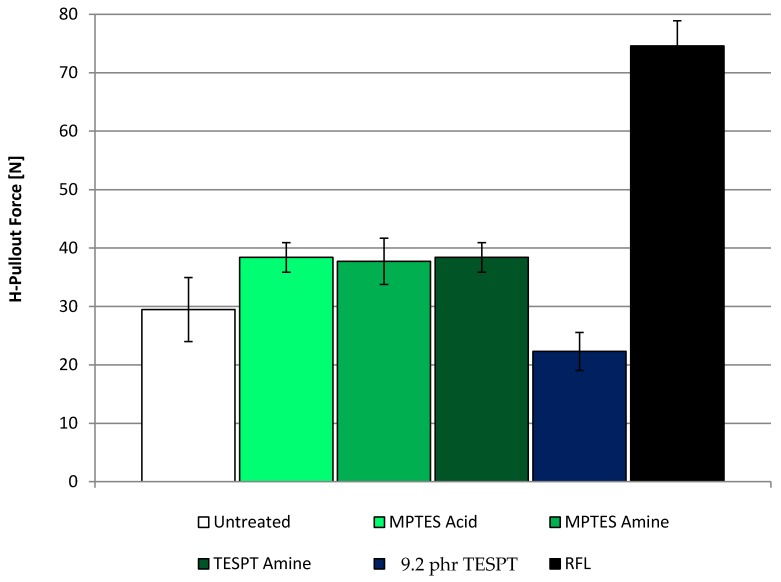
H-pullout results for TEOS-plasma-coated polyester cords.

Like for rayon, the results for the PET cords did not show a significant effect on the adhesion strength. The results of the experiments are illustrated in Figure 7 and were done under the same plasma treatment settings as used for rayon cord:

- Cord speed: 10 m/min
- TEOS flow rate: 150 g/h
- Evaporation temperature: 200 °C
- Pre-drying of cord: no

Clearly, the TEOS coating itself does not provide enough OH groups as seen from the test specimen with 9.2 phr TESPT incorporated into the compound. The adhesion value is even lower than the one of the untreated sample. The plasma treatment did not increase the number of hydroxide groups, therefore the TESPT present in the rubber compound was not able to react with any groups on the cord surface and the interaction with the cord surface was low. This is in contrast with the rayon results, as that material showed better values with this treatment. The PET results achieved with the type I bath dipping process (green bars) are better. Here the PET cord reaches even higher H-pullout forces than the comparable rayon cord. However, also the untreated PET cord shows better adhesion than the corresponding rayon test specimen. 

To achieve further improvements with this type of treatment, it is necessary to create functional groups on the cord surface, so that the coupling agent can react with them. An approach is to introduce more hydroxyl groups by using demineralized water as precursor. The expected effect is that additional reactive OH groups on the outside of the cord surface will form, and thus be available for silanization with coupling agents. The original OH groups of the polyester fibers are directed into the fiber structure and, thus, sterically hindered.

### 3.3. Applying H_2_O as a Precursor Instead of TEOS

The advantages of water over TEOS for activation of the cord surface are significant: In addition to the fact that it delivers an extra amount of reactive hydroxide groups, it does not show the drawbacks such as health risks, smell, and high costs. The evaporation temperature of the relatively small water molecule is 100 °C, much lower than for most other precursors. This makes it from a technical point of view easier to evaporate a sufficient amount of precursor before it enters the plasma jet. During the plasma treatment, no overpressure was applied; in fact the chamber was kept open to decrease the heat buildup and its negative effect on the cord. The dips with which the samples described in Table 4 and Table 5 were treated, had the following recipe:

- 95 mL Ethanol
- 5 mL H_2_O
- 5 wt% TESPT
- 2 wt% acetic acid

This solution was stirred for 2 h before usage. The series of experiments discussed in this section were done with the following plasma treatment settings:

- Cord speed: 10 m/min
- Evaporation temperature: 110 °C
- Pre-drying of cord: no

Flow rate (FR) and ionization gas (ION) were variable and are mentioned individually in Table 4, in which also the results for the PET test series are listed. Interestingly, with air as ionization gas there was no improvement over the previous TEOS trials; neither with the dipping method nor with the incorporation method. When nitrogen gas was applied instead of compressed air, a significant improvement with the incorporation method (compound with 9.2 phr TESPT) was found: an increase from about 40 N to 60 N of H-pullout force was measured.

The results for rayon are shown in Table 5: The trend is similar to the one seen for PET; the nitrogen gas also resulted in an improvement in adhesion. However, the effect is less prominent, and the highest values of pullout force are lower than the ones achieved with the sulfur precursor in earlier work [33]. In order to explore the potential of this treatment, a design of experiments (DoE) setup for the nitrogen/H_2_O treatment was performed. The DoE was focused on the dip properties as given in Paragraph 2.

These results of this study are gathered in Table 6.

**Figure 8:**
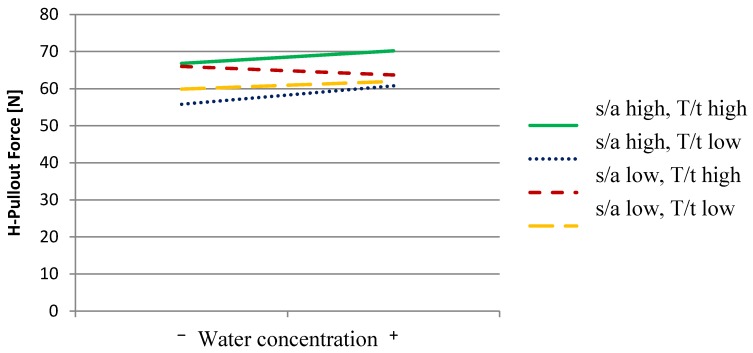
Effect of water content in the dip on adhesion. (s/a: silane (TESPT)/acid, T/t: temperature/time).

**Figure 9:**
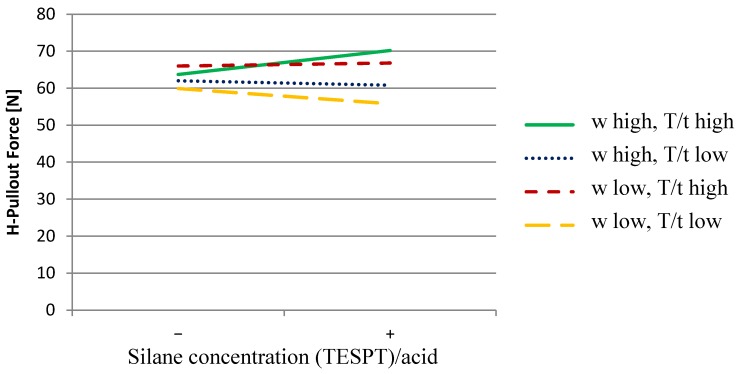
Effect of TESPT and acid content in the dip on adhesion. (w: water content, T/t: temperature/time).

**Figure 10:**
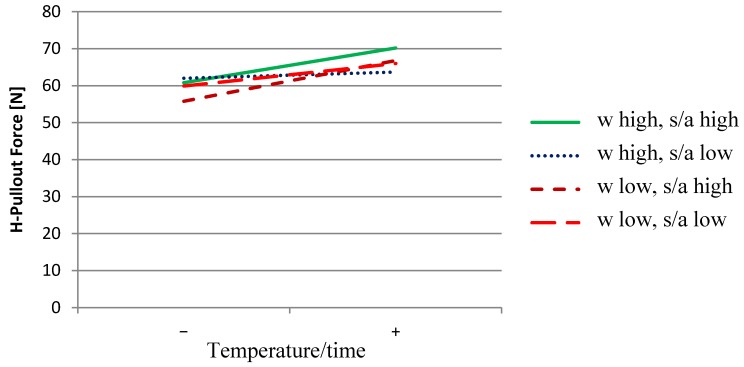
Effect of temperature and time on adhesion. (w: water content, s/a: silane (TESPT)/acid).

Figure 8, Figure 9 and Figure 10 feature each one parameter of the DoE experiments to highlight the effect it has on the other parameters in each possible configuration. One remarkable result is that the center point (CP) listed in Table 6, has the lowest adhesion values of all configurations. In Figure 8, the amount of water in the dip solution and its effect is shown. The samples with a plus of TESPT/acid benefit from additional water in the dip, while those with less TESPT/acid are not much affected. This is not surprising, as a higher amount of TESPT consequently requires more OH groups for silanization. If more TESPT is present, it will benefit from more OH groups independent of the temperature settings. Figure 9 shows the effect of TESPT and acetic acid on the silanization process. Improvements in adhesion are achieved when a high amount of OH groups is available (high water concentration) and the temperature is high enough. Otherwise it has no or even a negative influence on the performance. Finally, temperature and time effects are shown in Figure 9. Clearly, higher temperatures, namely 120 °C, in a shorter time interval (2 min) are more efficient than lower temperatures (60 °C) during a longer interval (120 min). The samples with more TESPT benefit the most from the elevated temperatures, but the trend is overall positive.

As a general conclusion it can be stated that the best adhesion results are achieved when high concentrations of water, TESPT and acid are used, and when the reaction is performed at higher temperatures and for longer times. 

## 4. Discussion

For rayon, the adhesion achieved by a silica coating followed by a silane-dip turned out to be rather low compared to RFL-treated cords as well as compared to plasma treatment with a precursor featuring a sulfur-moiety [33]. Two explanations are possible: Either the coating with TEOS itself was unable to deposit properly on the cord, or the deposited layer did not have enough OH groups to allow a strong interaction with the coupling agents. The results with the coupling agents incorporated in the matrix compound were comparable and independent on the TESPT concentration in the elastomer matrix, indicating that the silane as such is not the limiting factor. The fact that dipping of untreated cords results in the same range of adhesion values showed that the silica-deposition did not activate the cord surface for a reaction with the silane. 

For PET cords, the deposition of silica followed by a type I bath dipping resulted in higher adhesion than the comparable treatment of rayon cords. This difference between the two cord types is opposite to what was expected: less interaction of the inert PET cord compared to rayon. However, compared to the RFL coating, the adhesion is still considerably lower. 

Plasma activation with water using nitrogen as ionization gas gave significant improvements: the adhesion force increased to approximately 60 N; the reference RFL treatment adhesion strength was 83 N. This clearly indicates that this activation is very efficient. A possible explanation for the high efficiency is that the fragmentation voltage of nitrogen is higher compared to air, resulting in a higher energetic plasma. As PET is a very inert polymer, the additional energy of the nitrogen plasma very probably helps to activate the PET surface and to deposit hydroxylic groups.

For rayon, the adhesion achieved with water and nitrogen plasma treatment and silane-dipping was rather low compared to earlier results. The potential of this method was, therefore, further elucidated in a DoE-approach for the dipping and silanization steps. All investigated variables, concentrations as well as processing parameters, resulted in an improvement in adhesion, up to a level of 70 N. This means that the adhesion level of RFL treated cords was almost reached. Additionally, the still increasing trend for the different parameters shows that there is still room for further improvement.

## 5. Conclusions

Adhesion promotion for reinforcing cords in elastomers by plasma treatment is very promising and has potential to replace the conventional method of RFL coating. Creation of surface hydroxyl groups by a plasma treatment with water turned out to be more efficient than a deposition of silica by plasma-fragmentation of TEOS. The latter did not contain the required concentration of functional groups for a sufficient reaction with the coupling agents. 

By the use of demineralized water as precursor followed by a silane dip, a novel promising route was taken, which has additional advantages like absence of toxic chemicals or reaction products, ease of handling, and no environmental burden. This technology resulted in adhesion values of 72% of the values reached for RFL-treated PET cords. The adhesion levels measured for water-plasma treatment of rayon parameters found in this study were lower than the ones found in earlier studies with sulfur-containing precursors, but a DoE approach revealed that there is additional potential for this material. This method is most promising for PET, but further optimization is necessary.

## Figures and Tables

**Table 1:** Plasma coating settings for TEOS.

Frequency (kHz)	Actual Voltage (V)	Actual Current (A)	Calculated Power (kW)
25	296	12	3.5

**Table 2:** Compound for cord adhesion tests.

Ingredients	Specification	phr
NR	Standard Vietnamese Rubber SVR5	60.0
BR	Buna cis-132 (98% cis), Styron	20.0
SBR	Kralex 1723 (with 37.5 phr extender oil TDAE), Synthos	27.5
N-660	Reinforcing carbon black	40.0
Zinc oxide	Activator	3.0
Stearic acid	Activator	2.0
Naphthenic oil	Nytex 840, Nynas	13.0
TMQ	Antioxidant (2,2,4-trimethyl-1,2-dihydroquinoline)	0.5
6PPD	Antioxidant (octylated diphenylamine)	1.0
MBTS	Accelerator (2-Mercaptobenzothiazole disulfide)	0.1
TBBS	Accelerator (n-tert-butyl-2-benzothiazole sulfenamide)	0.7
S OT20	Insoluble sulfur	2.8
PVI	Scorch inhibitor (n-cyclohexylthio phthalimide)	0.1

**Table 3:** Dipping recipes used for rayon and PET cords with TEOS coating (TESPT as well as MPTES used for Bath I; TESPT for Bath II).

Ingredients	Bath I	wt%	Bath II	wt%
**Ethanol**	50.0 g	92.9	22.2 g	80.0
**Demin. water**	2.6 g	4.9	4.0 g	14.4
**Coupling agent**	1.1 g	2.0	1.0 g	3.6
**Catalyst**	0.1 g	0.2	0.6 g	2.0
**Total**	53.8 g	100.0	27.8 g	100.0

**Table 4:** H_2_O plasma-treated PET cord.

PET	Conditions		H-Pullout Force [N]		
Sample	ION	FR [g/h]	DIP 1	DIP 2	Incorp.
H_2_O	Air	300	42.1	40.5	40.0
H_2_O	Air	400	40.7	42.8	40.2
H_2_O	N2	400	-	-	59.8
RFL	-	-	-	-	83.0

**Table 5:** H_2_O plasma-treated rayon cord.

Rayon	Conditions		H-Pullout Force (N)	
Sample	ION	FR (g/h)	DIP	Incorp.
H_2_O	Air	300	45.1	44.8
H_2_O	Air	400	41.5	48.8
H_2_O	N2	400	DoE*	54.1
RFL	-	-	-	75.0

* Design of Experiments, see Table 6.

**Table 6:** DoE results for rayon cord.

DoE	H-Pullout Force (N)	
[A/B/C]	Value	Standard Deviation
CP	54.5	7.9
+++	70.2	6.0
+−+	63.7	5.3
−++	66.8	6.6
−−+	66.0	6.2
++−	60.8	4.2
+−−	62.0	6.4
−+−	55.8	4.8
−−−	59.9	5.3

## References

[1] Charch W.H., Buffalo N.Y., Maney D.B. (1938). Laminated Structure and Method for Preparing Same. U.S. Patent.

[2] Wennekes W.B., Datta R.N., Noordermeer J.W.M. (2007). Mechanistic investigations into the Adhesion between RFL-treated cords and rubber. Part I: The influence of rubber curatives. Rubber Chem. Technol..

[3] Wennekes W.B., Datta R.N., Noordermeer J.W.M. (2007). Mechanistic investigations into the Adhesion between RFL-treated cords and rubber. Part II: The influence of the Vinyl-Pyridine Content of the RFL-latex. Rubber Chem. Technol..

[4] Wennekes W.B., Datta R.N., Noordermeer J.W.M., Elkink F. (2008). Fiber adhesion to rubber compounds. Rubber Chem. Technol..

[5] Bekaert, Cordenka, Kordsa Introduction to tire reinforcement materials. Reader provided for a course given at the Tire Technology Conference, Tyre Technology Expo.

[6] Pocius A.V. (2012). Adhesion and Adhesives Technology.

[7] Franta I. (1989). Elastomers and Rubber Compounding Materials.

[8] Shishoo R. (2008). Textile Advances in the Automotive Industry.

[9] Mondadori N.M.L., Nunes R.C., Bresciani Canto L., Zattera A. (2012). Composites of recycled PET reinforced with short glass fiber. J. Thermoplast. Compos. Mater..

[10] Wootton D.B. (2001). The Application of Textiles in Rubber.

[11] (2012). Formaldehyde Registration Dossier.

[12] Cevahir N.N., Acar A.E., Bas S. (2014). A Dipping Material for Cord Fabrics and a Production Method Thereof. Patent.

[13] Solomon T.S. (1989). Two-Step Process for Dipping Textile Cord or Fabric and Resorcinol/Formaldehyde-Free Composition Used Therein. Patent.

[14] Bas S., Ergun B. (2014). A Method for Producing Fiber Reinforced Rubber Comprising Unsaturated Bonds. Patent.

[15] Lawton E. (1974). Bonding of Poly (Ethylene Terephthalate) Induced by Low-Temperature Plasmas. U.S. Patent.

[16] Sharma Satish C. (1984). Adhesion of Aramid Cords to Rubber. U.S. Patent.

[17] Shuttleworth D., Mowdood S.K. (1991). Process for the Surface Treatment of Polymers for Reinforcement-to-Rubber Adhesion. U.S. Patent.

[18] Shuttleworth D., Mowdood S.K. (1994). Process for the Surface Treatment of Polymers for Reinforcement-to-Rubber Adhesion. U.S. Patent.

[19] Morin B.G., Maria M.D.F. (2000). Vinyl Compound Plasma Pre-Treatment for Promoting the Adhesion between Textiles and Rubber Compounds. U.S. Patent.

[20] Parker D.K., Shuttleworth D. (1996). Process to Produce Reinforced Tire Cords with Plasma Treated Fibers Incorporated into a Rubber/Blocked Isocyanate Composition. U.S. Patent.

[21] Rodewald S., Siffer F.G.A. (2011). Plasmapolymerisierungsdüse, Luftplasmasystem damit und Verfahren zur Abscheidung eines Polymermaterials. Patent.

[22] Siffer F.G.A., Gillick J.G. (2011). Atmospheric Plasma Treatment of Reinforcement Cords and Use in Rubber Articles. U.S. Patent.

[23] Siffer F.G.A., Gillick J.G. (2014). Atmospheric Plasma Treatment of Reinforcement Cords and Use in Rubber Articles. Patent.

[24] Siffer F.G.A., Hebelman J.B., Gillick J.G., Gersman M.L., Chandra D., Qu D., Botts B.P., Vangeneugden D.L., Verheyde B., Van Hoof E.C.M. (2018). Atmospheric Plasma Treatment of Reinforcement Cords and Use in Rubber Articles. U.S. Patent.

[25] Ueno K., Sugimotot H. (1986). Method for Treating Fibers. U.S. Patent.

[26] Krump H., Hudec J., Jasso M., Dayss E., Crimmann P. (2004). Surface modification of PET by two different types of plasma reactors. Kautsch. Gummi Kunstst..

[27] Janypka P., Suriova V. (2006). Method of Treatment of Textile Reinforcing Materials to Increase Their Adhesion to Rubber Compound. Patent.

[28] Mihalik P., Suriova V. (2006). Device for Manufacturing Rubberized Fabric Cord, Suitable to Be Used as Wound Overlapping Breaker. Patent.

[29] Tonon C., Grimberg G. (1999). Process for Treating a Body of Stainless Steel so as to Promote Its Adherence to a Rubber Composition. Patent.

[30] Michelin. https://www.michelin.in/auto/home-auto/innovation.

[31] Luginsland H.-D. (2000). Reactivity of the sulfur chains of the tetrasulfane silane Si69 and the disulfane silane TESPD. Kautsch. Gummi Kunstst..

[32] Hunsche A., Görl U., Koban H.-G., Lehmann T. (1998). Investigations on the reaction silica/organosilane and organosilane/polymer-Part 2. Kinetic aspects of the silica-organosilane reaction. Kautsch. Gummi Kunstst..

[33] Louis A., Noordermeer J.W.M., Dierkes W.K., Blume A. Plasma modification of polymeric single end cords as an alternative to RFL treatment. Proceedings of the Fall 192th Technical Meeting of the Rubber Division ACS.

